# Relative Enthalpy of Solid Beryllium Aluminate (Chrysoberyl), BeO · Al_2_O_3_, from 1175 to 2025 K, and of Liquid Beryllium Aluminate from 2170 to 2350 K*

**DOI:** 10.6028/jres.080A.012

**Published:** 1976-02-01

**Authors:** S. Ishihara, E. D. West

**Keywords:** Beryllium aluminate, chrysoberyl, drop calorimetry, enthalpy measurements, high temperature calorimetry, specific heat, thermodynamic properties

## Abstract

The relative enthalpy of solid beryllium aluminate BeO · Al_2_O_3_ from 1180 to 2025 K and liquid beryllium aluminate from 2170 to 2350 K was measured by “drop” calorimetry using an adiabatic “receiving type” calorimeter. The thermodynamic functions from 1175 to 2025 K and the enthalpy of melting at 2146 K are reported.

## 1. Introduction

Naturally occurring BeO · Al_2_O_3_ (chrysoberyl) has variable and appreciable amounts of impurities such as Cr, Fe, Si, Na, Li, K, and Ti [3, 4, 5].^1^ Little or no accurate work has been done on the thermodynamic properties of this material in its natural state. The heat capacity of synthetic 1:1 beryllium aluminate from 16 to 380 K and the relative enthalpy from 273 to 1173 K have been previously measured at the National Bureau of Standards [1, 2]. The present work has been undertaken to extend the range of accurate thermodynamic data on this material to near its melting temperature. BeO · Al_2_O_3_ is reported to melt at 2146 ± 10 K^2^ [6]. We have made some enthalpy measurements beyond this temperature in a welded capsule so that the enthalpy of melting can be estimated.

## 2. Experimental Procedures

### 2.1. Sample

The BeO · Al_2_O_3_ sample used in this work was prepared by Semi-Elements Inc. of Saxonburg, Pa.,^3^ by arc-fusion of a stoichiometric mixture of high purity powdered BeO and Al_2_O_3_. The homogeneity of the lot has been adequately established and the sample is from the same lot as described in references [1, 2]. The fine material in the samples was removed by passing it thru a 16-mesh stainless steel screen. Occasional dark particles remaining on the screen were discarded.

After 2.5 years storage in a plastic bottle on a laboratory shelf the water content was determined to be 0.16 percent by the Microchemical Analysis Section of the National Bureau of Standards. The sample was heated to 1325 K in a carbon-hydrogen-nitrogen analyzer. The water in the sample was separated by a gas chromatographic column and then detected by thermoconductivity. This result is consistent with the 0.2 percent mass loss observed in our furnace under similar conditions.

A new spectrochemical analysis of the sample showed small differences from the earlier ones [1, 2]. The results of the analyses are tabulated in table 1. Six samples designated A, B, C, D, G, and H were used in this work of which C and D were not analyzed because they duplicate A and unexposed samples, respectively. The only significant difference between the exposed and unexposed samples is the small amount of container material found in the former. This could be due to abrasive action of the hard material on filling and emptying the container, chemical combination, or sublimation. After exposure to higher temperatures (1700 K and above) the surface of the crystals appeared slightly grayish and the fused sample showed patches of gray areas; however, no difference in chemical composition between the light and gray areas could be detected.

The samples are identified as in tables 1 and 2. A brief history of the samples is as follows:

A. Heated in an unsealed molybdenum container. Total mass loss in two experiments at 1700 K was 0.38 percent.
B. Heated in an unsealed molybdenum container. Mass loss on heating to 1700 K was 0.4 percent and additional loss on heating to 2000 K, 0.07 percent. There was no further significant change in mass.
C. Heated in an unsealed molybdenum container. Mass loss on heating to 1180 K was 0.25 percent with an additional loss of 0.1 percent on heating to 1700 K. There were no further significant changes.
D. Heated in an unsealed molybdenum container. Mass loss on heating to 1180 K was 0.24 percent.
G. Heated 6 hours in a platinum crucible in the flame of Meker burner at 1200 K before loading in a molybdenum inner container. During the sealing process by electron beam welding, the container lost 3.06 mg. Because of the extremely localized heating of the process, the mass loss was taken to be molybdenum. No further significant mass changes were observed.
H. Pretreated as in G but loaded in a tungsten inner container. During the welding process this container lost 2.70 mg. There were no further significant mass changes.

### 2.2. Containers

Tungsten and molybdenum, both 99.95 percent pure, were used as container materials. Two types of containers are used. The first type, which we call an outer container, is used at lower temperatures where sample mass losses are negligible. It is not sealed, so that the sample can be loaded into the container as small lumps and removed to determine a sample and container mass for each experiment. The second type, used at higher temperatures and throughout the liquid range, consists of the unsealed outer container and an inner container vacuum sealed by electron beam welding after adding a weighed amount of sample. This technique has the advantage that the mass of the inner container does not change and any significant changes in mass of the outer container can be accounted for experimentally. It has the disadvantage that the experiment gives the combined enthalpy of the sample plus the inner container, for which a correction must be made.

To determine this correction, the enthalpy of a molybdenum sample, machined from the same rod used for the inner container, was determined in the same series of measurements and in the same outer container, whenever the enthalpy of an inner container with sample was measured.

### 2.3. Enthalpy Measurements

The method of enthalpy measurements and the apparatus used in this work [15] will be described briefly. After the capsule (full or empty) has reached temperature equilibrium in the furnace it is lifted from the furnace into a “receiving” type adiabatic calorimeter operating near room temperature. If the position, flight time, shutter sequence, emissivity, and the furnace temperature are the same, the difference in heat energy received by the calorimeter from the full and empty capsule is the change in enthalpy of the contents. An automatic servo-regulated lifting device is used to keep the position and flight time of the capsule the same. The furnace is inductively heated and a Leeds and Northrup automatic optical pyrometer monitors and controls the temperature of the graphite core or the bottom of the capsule by sighting on it through a prism at the bottom end of the furnace. A number of changes have been made since the previous description of the apparatus [15] and are described as follows.

When the apparatus was moved from Washington, D.C. to Gaithersburg, Md. to a laboratory in which the temperature is regulated to ±1 K, the new environment eliminated much of the problem associated with the effect of room temperature on the automatic optical pyrometer. This effect was further reduced by replacing some temperature-sensitive germanium transistors with more stable silicon transistors.

A solid state reference voltage supply has replaced the mercury cell. The stability of the furnace temperature depends directly on the constancy of this supply. The variations in the furnace temperature are now so small that they have a negligible effect on the measured heat (< .02%).

For measurement of the temperature of the adiabatic calorimeter, a copper resistance thermometer wound noninductively and cemented with an epoxy resin in a groove in the copper block has replaced the platinum resistance thermometer. Comparisons made between the two thermometers over a period of 3 years indicated that the copper thermometer was stable within the imprecision of the measurement. Its resistance is about 80 Ω and the change in resistance per degree is about three times as large as the resistance change in the platinum thermometer. It also has the advantage of excellent thermal contact with the calorimeter so that its excess temperature due to heat generated by the thermometer current is estimated to be 6 × 10^−5^ K at 2 mA, compared to 2 × 10^−3^ K for the platinum thermometer. The change in excess temperature for ordinary variations in thermometer current is now negligible. Electrical calibrations of the adiabatic calorimeter conducted with the copper thermometer over a period of 2½ years in this laboratory now have an estimated standard deviation of 0.004 percent.

Recirculated chilled water at 10.5 °C is now used to cool the induction heater and the furnace ends, and has replaced the tap water and water economizer used in the old location. Since the power to maintain the furnace at a preset temperature does not change during the day, the temperature gradient in the furnace is more stable.

The experimental procedure is now designed to account for mass gains of the outer container. Three or four individual experiments are made in a day and are ordered so that the first and last experiments are duplicates. When the mass of the outer container changes between the first and last experiments, any difference in the total heat for these experiments, after appropriate corrections, is assumed to be proportional to the mass gain and intermediate experiments are corrected according to the mass gain in each. In practice, the correction factor in joules per milligram is determined by plotting determinations for all experiments and drawing a smooth curve through them. Above 2000 K the correction factor is virtually constant. The magnitude of this correction ranges from negligible at temperatures below 1600 K to 5 percent of the enthalpy of the liquid BeO · Al_2_O_3_ at the higher temperatures. (The liquid sample is about one-half the mass of the solid sample.) Masses are now determined after each individual experiment, which considerably slows the work but permits the empirical correction for each experiment. The advantage of the empirical correction is that it does not assume any particular chemical process for the mass gain.

The chronological sequence of the furnace temperatures for the measurements on the solid work was selected randomly. The measurements in the premelting region and in the liquid phase were run on later dates after the machining, engineering, and welding problems were solved for the inner container.

The sequence for the empty and full experiments was staggered in order not to introduce a bias due to gradual change in the furnace insulation.

### 2.4. Results

Presented in table 2 are the results of the individual experiments. The quantity of heat delivered to the calorimeter includes the small corrections for the enthalpy of the container and its contents between the final calorimeter temperature and 298.15 K. These corrections were at most 2 percent of the measured heat so that the effect of the uncertainty in the data used in making them [1,8, 9, 10] is negligible. Wherever the correction data were presented in calorie units, they were converted to joules by multiplying by the conversion factor 4.1840. In column 2 the larger figures for a given day are the measured heats for a full capsule and the smaller figures refer to the empty capsule. To account for the tungsten inner container in the premelting and liquid range the correction data were taken from reference [7]. The enthalpy for the molybdenum inner container was measured along with the BeO · Al_2_O_3_ when this material was used, and is tabulated in the table along with the other experiments for the day. Weighing was done in air and the correction for buoyancy is based on a density of 3.71 gm/cm^3^ for chrysoberyl. The temperature scale is IPTS 1968 [11, 12].

Equation (1) (see below) represents the enthalpy of solid BeO · Al_2_O_3_; it was computed by the method of least squares [13]. Since the uncertainty in the temperature is greater at higher temperatures, the results of individual experiments were weighted inversely to the magnitude of the molar enthalpy. The coefficients of eq (1) were calculated using the results reported herein with the results of Ditmars and Douglas [2] and Furukawa and Saba [1]. The joining of the data sets is shown in figure 2. Equation (1) and the coefficients of the equation are as follows:

$$H_{T}-H_{298.15}=(A/3)T^{-3}+(B/2)T^{-2}+CT^{-1}+D\;\ln\;(T)+ET+(F/2)T^{2}+(G/3)T^{3}+(H/4)T^{4}+(I/5)T^{5}+J\;1180<T<2030$$ (1)

^4^

where *H_T_* − *H*_298.15_ = relative enthalpy in kJ · mol^−1^

T = temperature (K)
A = −1.846_00_ × 10^7^
B = 8.189_59_ × 10^5^
C = −1.438_99_ × 10^4^
D = −1.248_90_ × 10^2^
E = 5.066_04_× 10^−1^
F = −5.069_47_ × 10^−4^
G = 4.592_67_ × 10^7^
H = −2.141_71_ × 10^−10^
I = 4.075_17_ × 10^−14^
J = 623.2_97_

The estimated standard deviation of the observed points from the smoothed curve is ±0.142 percent for the range 1180 to 2100 K.

**Figure 1:**
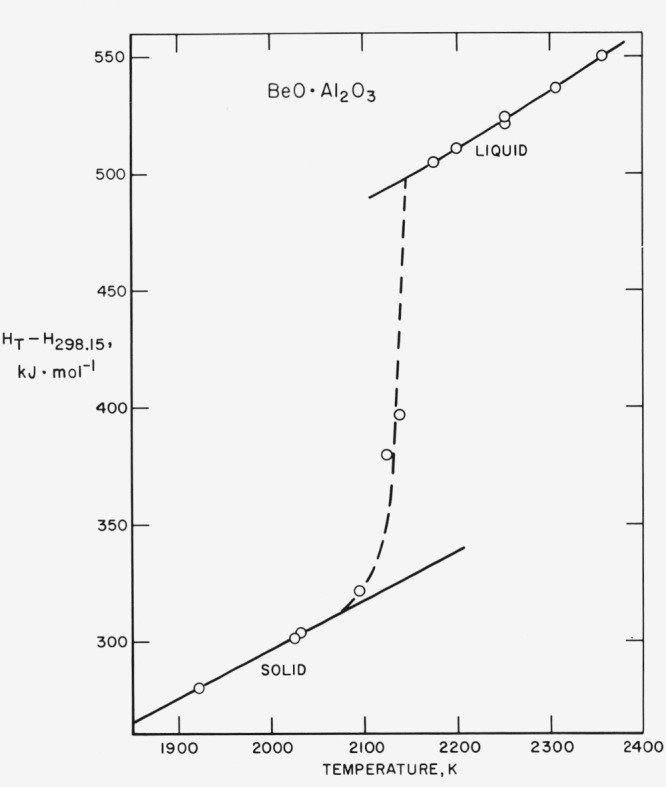
Enthalpy relative to 298.15 K measured near the melting temperature.

Beyond 2125 K and up through 2137 K premelting of chrysoberyl is evidenced by the sharp departure of the observed enthalpies from the smoothed solid and liquid curves. (See fig. 1.) There is a gradual change of the dimensions of the inner container in this range as well as at the higher temperatures as the sample is cycled to room temperature. Presumably, this is caused by the rapid expansion and contraction of chrysoberyl. The tungsten container ultimately fractured thus limiting the number of experimental points.

For the liquid range the enthalpy of chrysoberyl can be represented by the equation:

$$\begin{array}{l} H_{T}-H_{298.15}=7683_{94.}-462.4_{16}T+0.1568_{10}T^{2}\\ \;J\cdot {\text{mol}}^{-1}\;\text{for}\;2175<T<2355 \end{array}$$ (2)

The root mean square deviation of a single determination (3 individual experiments) from the smoothed curve is ±0.16 percent.

Taking 2146 K on the International Practical Temperature Scale of 1968 to be the melting temperature [6], the enthalpy of fusion is calculated to be 171.5 kJ • mol^−1^ by extrapolating eqs (1) and (2) to this temperature. An uncertainty of ± 10 K in the melting temperature will have an effect on the enthalpy of fusion no larger than ±0.10 percent. Extrapolation of the smoothed curves will introduce an uncertainty estimated to be ±0.3 kJ • mol^−1^ at 2146 K.

A few measurements on a sample of molybdenum from the same rod used for the inner container have been made at 2033, 2095, 2125, 2200, and 2254 K. The results are listed in table 2 along with those for chrysoberyl. These experiments were carried out simultaneously because the best data found in literature [14] claim a maximum error of ±1.2 percent. The results obtained are within 0.48 percent of the reported values.

## 3. Discussion

### 3.1. Polynomial Curve Fitting for the Solid Phase

The usual method of fitting the enthalpy measurements to an empirical equation by the method of least squares [13] was employed to establish how well we can expect the curve to represent the observed data from 1180 to 2030 K. The estimated standard deviation of the observed enthalpies of the solid phase from a curve fit (not shown) of this work alone is ±0.10 percent.

Since accurate thermodynamic data on this material at lower temperatures are available from earlier works [1, 2], it was decided to make use of their results to extend the range down through 273 K. Certain conditions must be set to do this. First is that the heat capacity of the substance is well behaved and normal throughout the region of study. There is no evidence that this is not so. The amount of impurities is small. The BeO and BeO · 3Al_2_O_3_ phases were not detected. Lang, Fillmore, and Maxwell [6] redetermined the equilibrium diagram of the beryllia-alumina system and their results indicate no phase transition in this temperature range. References [1, 2] report no unusual behavior in the heat capacities at lower temperatures.

Second is that the enthalpy and heat capacity curves are smooth functions throughout this range. This is reasonably assured if the first condition is satisfied and if all the energy, temperature and mass measurements are compared to the same standards during calibration and observations.

A third condition set on the curve fit is that the equation justifies the accuracy of measurements for the three studies at different temperature ranges.

**Figure 2:**
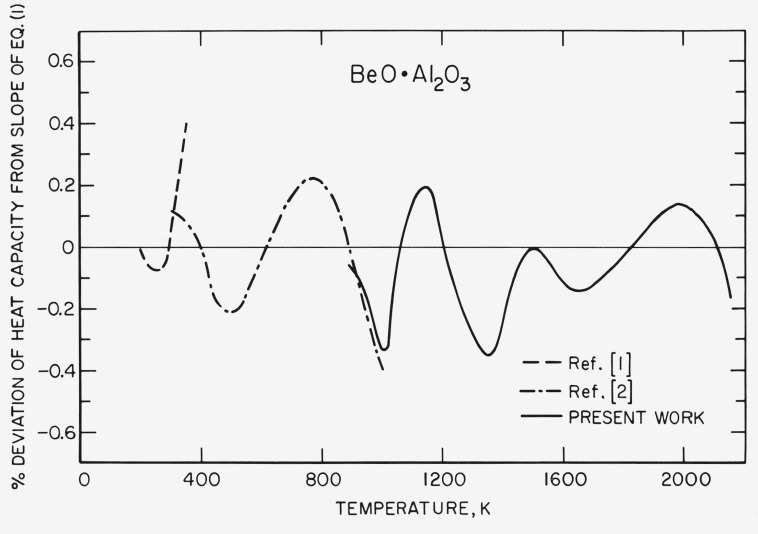
The effect of “smooth joining” of the enthalpies of 1:1 *BeO · Al_2_O_3_* on the heat capacities.

Equation (1) can be used down to 273 K with little or no loss in accuracy. Figure 2 compares the values of the heat capacities from equation (1) with the present work and with those reported in references [1, 2].

### 3.2. Liquid Phase and the Heat of Fusion

Equation (2) represents the relative enthalpy of chrysoberyl in the liquid phase for the range 2175 to 2355 K. A polynomial of 2d degree was selected having least squares deviation and 95 percent confidence limits because a straight line representation (usually assigned to enthalpies for such a small temperature range and small number of observations) does not adequately represent the observed values.

The uncertainty limits on the heat of fusion of ±3.5 kJ • mol^−1^ includes 1.5 kJ • mol^−1^ for the extrapolation error of the solid curve, 1.5 kJ • mol^−1^ for the extrapolation error of the liquid curve, and an error of 0.5 kJ • mol^−1^ due to the combined effect of the temperature uncertainty and of the compromise in merging our results with lower temperature work.

Not enough observations in the premelting stage, 2025 < *T* < 2150, were made to give a definitive explanation to its cause except to state that molybdenum and tungsten, the inner container, were detected in the post-measurement spectral analyses of the samples.

### 3.3. Errors

There are three principle sources of error involved in the present calorimetric method —one, the temperature measurements; two, the energy measurements; and three, the sample purity and behavior.

The stability of both the solid state circuitry and standard reference lamp in the automatic optical pyrometer was examined by repeated calibrations (5) during the 4 years of usage. The uncertainty ranged from ±4 K at 3000 K to ±0.7 K at 1325 K (with the usual precautions in frequent checks on optical alignment, focus, and cleanliness). This compares closely with the ±0.1 percent deviation that we obtained in the curve fitting.

The operational procedure was designed so that we could evaluate the imprecision of the energy measurements for the solid range. This turned out surprisingly simple because the furnace temperature can be controlled to ±0.02 percent during a day’s work. By making duplicate observations on the same day at one furnace temperature, the imprecision of the entire temperature range of the solid phase was expressed as:

$$S_{p}^{2}=\frac{1}{2n}\sum_{i=1}^{n}d_{i}^{2}$$

where

*S_p_*: = estimated imprecision
*n*: = number of observations made at different temperatures
*d_i_*: = percent difference of the two enthalpy measurements at a given temperature.

We calculate *S_p_* = ±0.046 percent.

Spectrochemical analyses of exposed and unexposed chrysoberyl show very little difference in metallic impurities. Molybdenum found in the exposed sample (less than 0.1%) can only come from the container. If we assume that the atomic specific heat is the same for the elements involved, the error in the enthalpy would be less than 0.002 percent. Although this small amount introduces a bias to the overall measurement, corrections were not applied because the uncertainty is overshadowed by the greater uncertainty in the furnace temperature, because four samples were used making it difficult to estimate the amount of molybdenum impurity present in each experiment, and because the weight losses reported in section 2.1 might not be entirely due to evaporation of chrysoberyl as we have assumed.

Considering all the errors that were discussed we believe that other systematic errors encountered in this measurement are negligible compared to the estimated uncertainty of ±0.32 percent in the relative enthalpy of the solid phase.

The thermodynamic functions (see table 3) were prepared by routine smoothing and numerical integration of the heat capacities from eq (1). The enthalpy and entropy values at 273.15 K are from references [1, 2].

## Figures and Tables

**Table 1:** Impurities detected in the spectrochemical analyses of *BeO · Al_2_O_3_*

Mass %						

	Ref. [1, 2]	Unexposed	A	B	G	H
	
Ag	< 0.0001	—	—		< 0.001	—
Ba	—	< 0.001	< 0.001	—	< .001	< 0.001
Ca	.001–.01	.001–.01	.001–.01	<0.001	<.001	< .001
Cr	—	< .001	< .001	—	.001–.01	.001–.01
Cu	.01–.1	.001–.01	< .001	.001–.01	.001–.01	.01–.1
Fe	.001–.01	.01–.1	.01–.1	.01–.1	.001–.01	.01–.1
Mg	.001–.01	.01–.1	.01–.1	.01–.1	.01–.1	.01–.1
Mn	—	< .001	< .001	—	<.001	< .001
Mo	—	—	.01–.1	.01–.1	.001–.01	—
Na	—	—	—	—	.01–.1	.01–.1
Ni	.01–.1	—	—	—	—	—
Pb	< .001	—	—	—	—	—
Si	< .01	.01–.1	.01–.1	.001–.01	.001–.01	.001–.01
Sn	< .001	—	—	—	—	—
Sr	—	—	—	—	.001–.01	.001–.01
V	.001–.01	—	—	—	—	—
W	—	—	—	—	—	< .001

**Table 2:** Enthalpy measurements of solid, premelting and liquid *BeO · Al_2_O_3_^a^*

Furnace temp. (K), date, and container material	Heat to calorimeter at 298.15 K Joules	Millimoles BeO · Al_2_O_3_ and sample designation	Enthalpy *H_T_* – *H*_298.15_ KJ · mol^−1^	% Deviation from smoothed curve
1182.2	4549.9	15.456 D	139.62	0.008
2–6–67	2391.9			
Molybdenum	2391.1			
	4677.8	16.370 B	139.69	.043
1182.2	4674.4	16.370 B	139.52	−.080
2–3–67	2390.5			
Mo	2390.5			
	4675.8	16.370 B	139.60	−.021
1308.4	2760.7			
2–21–67	5417.4	16.370 B	162.29	−.078
Mo	5418.7	16.370 B	162.38	−.024
	2760.6			
1411.3	6039.0	16.369 B	181.21	−.012
2–13–67	3072.7			
Mo	3072.5			
	6038.5	16.369 B	181.91	−.023
1411.2	6154.3	17.025 C	181.07	−.076
1–30–67	3071.6			
Mo	3071.3			
	6155.7	17.022 C	181.20	−.005
1511.9	3376.7			
2–16–67	6650.6	16.369 B	200.01	.080
Mo	6651.5	16.369 B	199.93	.040
	3378.9			
1612.4	7272.5	16.368 B	218.18	−.229
2–27–67	3701.3			
Mo	3703.0			
	7276.5	16.368 B	218.31	−.168
1717.2	7935.1	16.477 B	239.33	.288
1–18–67	3991.6			
Mo				
1719.6	3973.0			
1–11–67	7945.0	16.603 A	239.23	.055
Mo	7945.4	16.603 A	239.21	.048
	3973.7			
1818.9	8601.2	16.369 B	258.28	−0.049
2–23–67	4373.4			
Mo	4374.8			
	8599.4	16.369 B	258.08	−.126
1923.0	4771.9			
3–1–67	9334.1	16.368 B	278.74	−.167
Mo	9336.0	16.367 B	278.95	−.090
	4770.4			
2026.7	10048.7	16.387 B	300.62	−.027
1–20–67	5122.4			
Mo	5120.2			
	10047.6	16.371 B	300.98	.094
2033.3	4330.3			
5–24–67	9419.7	10.830 G	303.72	.534
W, sample in sealed Mo container	b(8258.5) 4332.6	(76.1002)	(51.60)	
2095.4	4620.5			
6–5–67	9983.4	10.830 G	321.37	
W, Mo	4626.8			
	b(8744.6)	(76.1002)	(53.94)	
2125.2	4611.1			
5–29–67	10649.0	10.830 G	380.29	
W, Mo	b(8804.1)	(76.1002)	(55.11)	
	4609.8			
2137.3	5600.0			
7–17–67	12210.6	7.5154 H	396.68	
W, W	12229.2	7.5154 H	397.75	
	5610.6			
2177.1	5737.5			
7–26–67	13243.0	7.5154 H	504.69	−.04
W, W	13244.6	7.5154 H	504.91	0.00
2200.1	12494.9	10.8297 G	510.30	.05
6–7–67	b(9359.4)	(76.1002)	(57.94)	
W, Mo	4951.5			
	12497.3	10.8297 G		
2253.6	5243.6			
6–9–67	b(9802.6)	(76.1002)	(59.91)	
W, Mo	13002.8	10.8297 G	523.73	.20
2253.8	5365.6			
6–23–67	13174.9	7.5154 H	521.89	−.16
W, W	5357.6			
2307.49	13899.3	7.5154 H	535.96	−.07
7–7–67	5858.5			
W, W	13909.5	7.5154 H		
2355.0	6203.7			
7–11–67	14456.6	7.5154 H	549.23	.03
W, W	6190.6			

a Relative molecular mass is 126.9728; density of solid, 3.71 gm/cm^3^.

b Molybdenum experiment.

**Table 3:** Molar thermodynamic functions of chrysoberyl

Solid phase						

Mol = 0.1269728 KG 1 Cal = 4.1840 J						

*T*	*C_p_*	$\left(H_{T}^{0}-H_{0}^{C}\right)$	$\left(H_{T}^{0}-H_{0}^{C}\right)/T$	$\left(S_{T}^{0}-S_{0}^{C}\right)$	$-\left(G_{T}^{0}-H_{0}^{C}\right)$	$-\left(G_{T}^{0}-H_{0}^{C}\right)/T$
*K*	*J/K*	*J*	*J/K*	*J/K*	*J*	*J/K*
273.15	97.036	10558.	38.652	57.430	5129.1	18.778
275.00	97.688	10738.	39.047	58.087	5236.0	19.040
280.00	99.420	11230.	40.110	59.863	5530.8	19.753
285.00	101.11	11732.	41.165	61.638	5834.6	20.472
290.00	102.76	12241.	42.213	63.411	6147.2	21.197
295.00	104.37	12759.	43.253	65.181	6468.7	21.928
298.15	105.36	13090.	43.904	66.295	6675.8	22.391
300.00	105.94	13285.	44.285	66.948	6799.0	22.663
310.00	108.96	14360.	46.323	70.472	7486.1	24.149
320.00	111.83	15464.	48.325	73.977	8208.4	25.651
330.00	114.56	16596.	50.292	77.460	8965.6	27.168
340.00	117.16	17755.	52.221	80.919	9757.5	28.699
350.00	119.63	18939.	54.112	84.351	10583.	30.240
360.00	121.98	20147.	55.964	87.755	11444.	31.790
370.00	124.21	21378.	57.779	91.127	12338.	33.348
373.15	124.89	21770.	58.343	92.183	12627.	33.840
380.00	126.34	22631.	59.555	94.468	13266.	34.913
390.00	128.36	23904.	61.294	97.776	14228.	36.482
400.00	130.28	25197.	62.995	101.05	15222.	38.056
410.00	132.11	26510.	64.658	104.29	16249.	39.632
420.00	133.86	27839.	66.285	107.49	17308.	41.209
430.00	135.52	29186.	67.876	110.66	18398.	42.788
440.00	137.11	30550.	69.432	113.80	19521.	44.366
450.00	138.62	31928.	70.953	116.90	20674.	45.944
460.00	140.07	33322.	72.440	119.96	21859.	47.519
470.00	141.45	34730.	73.894	122.99	23073.	49.093
480.00	142.77	36151.	75.315	125.98	24318.	50.664
490.00	144.04	37585.	76.705	128.94	25593.	52.231
500.00	145.25	39031.	78.064	131.86	26897.	53.794
550.00	150.58	46432.	84.422	145.96	33846.	61.538
600.00	154.97	54074.	90.124	159.26	41479.	69.133
650.00	158.53	61916.	95.257	171.81	49759.	76.553
700.00	161.75	69928.	99.897	183.68	58649.	83.785
750.00	164.45	78084.	104.11	194.94	68117.	90.823
800.00	166.83	86368.	107.96	205.63	78133.	97.667
850.00	168.95	94763.	111.49	215.81	88671.	104.32
900.00	170.87	103260.	114.73	225.52	99706.	110.78
950.00	172.61	111840.	117.73	234.80	111210.	117.07
1000.00	174.20	120510.	120.52	243.70	123180.	123.18
1050.00	175.67	129260.	123.11	252.23	135580.	129.12
1100.00	177.03	138080.	125.53	260.44	148390.	134.91
1150.00	178.30	146960.	127.80	268.34	161610.	140.54
1173.15	178.85	151100.	128.80	271.89	167870.	143.10
1200.00	179.48	155910.	129.93	275.95	175220.	146.02
1250.00	180.60	164910.	131.93	283.30	189200.	151.37
1300.00	181.67	173970.	133.82	290.40	203550.	156.58
1350.00	182.71	183080.	135.62	297.28	218240.	161.66
1400.00	183.75	192240.	137.32	303.94	233270.	166.63
1450.00	184.80	201450.	138.94	310.41	248630.	171.47
1500.00	185.91	210720.	140.48	316.69	264310.	176.21
1550.00	187.10	220040.	141.97	322.81	280300.	180.84
1600.00	188.41	229430.	143.40	328.77	296590.	185.37
1650.00	189.83	238890.	144.78	334.59	313170.	189.80
1700.00	191.56	248420.	146.13	340.28	330050.	194.15
1750.00	193.52	258050.	147.46	345.86	347200.	198.40
1800.00	195.79	267780.	148.77	351.34	364630.	202.57
1850.00	198.45	277630.	150.07	356.74	382330.	206.67
1900.00	201.57	287630.	151.39	362.08	400300.	210.69
1950.00	205.23	297800.	152.72	367.36	418540.	214.64
2000.00	209.49	308160.	154.08	372.61	437040.	218.52
2050.00	214.44	318760.	155.49	377.84	455800.	222.34
2100.00	220.19	329620.	156.97	383.07	474820.	226.11
2146.00	226.25	339890.	158.38	387.91	492550.	229.52

$H_{0}^{C}$ and $S_{0}^{C}$ apply to the reference state of the solid at zero deg K. temperature scale is IPTS 1968.

## Footnotes

1 Figures in brackets indicate the literature references at the end of this paper.

2 IPTS 1968.

3 Certain commercial materials and products are identified in this paper to adequately specify the experimental procedure. Such identification does not imply recommendation or endorsement by the National Bureau of Standards.

4 In designates natural logarithm.

## References

[1] Furukawa GT, Saba WG (1965). J Res Nat Bur Stand (US).

[2] Ditmars DA, Douglas TB (1967). J Res Nat Bur Stand (US).

[3] Palache C, Berman H, Frondel C (1944). The System of Mineralogy.

[4] Apollonov VN (1967). Zap Vses Mineral Obshchest.

[5] (1962).

[6] Lang AM, Fillmore CL, Maxwell LH (1952). J Res Nat Bur Stand (US).

[7] West ED, Ishihara S (1968). National Bureau of Standards Report No 9803.

[b8-jresv80an1p65_a1b] 8Reilly, M. L., Furukawa, G. T., “Critical analysis of the heat capacity data of the literature and evaluation of thermodynamic properties of Cr, Mo, and W from 0 to 300 K, to be published.

[9] Starrett KF, Wallace WE (1958). J Am Chem Soc.

[b10-jresv80an1p65_a1b] 10Furukawa, G. T., private communication.

[11] (1969). The International Practical Temperature Scale 1968. Metrologia.

[12] Douglas TB (1969). J Res Nat Bur Stand (US).

[b13-jresv80an1p65_a1b] 13Natrella, M. G., Experimental Statistics, NBS Handbook 91.

[14] Kirillin VA, Sheindlin AE, Chekhovskoi VYa (1962). Int J Heat Mass Transfer.

[15] (1970). Precision Measurement and Calibration (Selected NBS Papers on Heat).

